# Comment on Małgorzata Krówczyńska and Ewa Wilk. Environmental and Occupational Exposure to Asbestos as a Result of Consumption and Use in Poland. *Int. J. Environ. Res. Public Health* 2019, *16*, 2611

**DOI:** 10.3390/ijerph17051662

**Published:** 2020-03-04

**Authors:** Beata Świątkowska, Wojciech Hanke

**Affiliations:** 1 The Reference Center for Asbestos Exposure & Health Risk Assessment, Department of Environmental Epidemiology, Nofer Institute of Occupational Medicine, 8 Teresy St., 91-348 Łódź, Poland; 2 Central Register of Occupational Diseases, Department of Environmental Epidemiology, Nofer Institute of Occupational Medicine, 8 Teresy St., 91-348 Łódź, Poland; wojciech.hanke@imp.lodz.pl

Krówczyńska M. and Wilk E. have recently published an article in the journal, entitled “Environmental and occupational exposure to asbestos as a result of consumption and use in Poland” [1]. The purpose of their study was to present asbestos issues in Poland with regard to the underestimation of environmental asbestos exposure. In the paper, environmental asbestos exposure was examined in relation to asbestos use and manufacturing, the measurements of asbestos fibre concentrations in the air, and the number of mesothelioma cases (MM). The authors explained in the Materials and Methods section that “all available data on MM cases were derived from the National Cancer Registry”. In their analysis, the authors reported the absolute number of MM cases in counties due to environmental and para-occupational exposure to asbestos (Figure 4, with detailed information by county). Unfortunately, it is not clear how the environmental history was collected, and how the determination is made that the disease is the result of environmental exposure. Attribution of cause of cancer (environmental, occupational, para-occupational) from the National Cancer Register, the source of cases for analysis provided by the authors, is not available.

The only available, reliable source on the origin of mesothelioma is occupational exposure data. In Poland, each case of diagnosed occupational disease is registered. Data on occupational diseases has been collected by the Nofer Institute of Occupational Medicine (NIOM) in Lodz in the Central Register of Occupational Diseases. Despite the non-use of asbestos in production in our country, there are still new cases of mesotheliomas among former asbestos workers. This situation is related to the specificity of the biological activity of asbestos, where the health consequences may manifest not only during the exposure but also many years after exposure cessation [2]. During the period 2001–2014, there were 2726 asbestos-related diseases, including 289 pleural or peritoneal mesothelioma patients [3,4,5]. The authors did not submit a request to provide extract data from the Central Register of Occupational Diseases. Detailed data, by county, are not available online.

Additionally, since 2000, former workers of asbestos-processing plants in Poland have been entitled to periodic medical examinations (medical check-ups) under the Amiantus Programme. The periodic medical examination programme is conducted so as to meet the statutory rights of the workers employed in 28 Polish asbestos-processing plants, which were listed in the Act of 19 June 1997 on the ban on the use of asbestos-containing products. The programme has increased the number of reported cases to the Central Register of Occupational Diseases and the detection of pathologies associated with asbestos exposure. 

In the Results section, the authors presented data from the Amiantus Programme—the number of surveyed people and the number of reported mesothelioma cases among former asbestos processing plant workers. Unfortunately, the presented data does not provide true information. According to the Reference Center for Asbestos Exposure and Health Risk Assessment, which is the programme coordinator, since launching the Amiantus Programme in 2013, the study covered 7374 people (not 7020, as presented by the authors), for whom a total of 29,333 medical examinations were performed and 58 diseases were diagnosed as mesothelioma (not 138, as presented by the authors). Until 2015, 68 cases of pleural mesothelioma have been diagnosed among former asbestos processing plants in Poland [5]. The program is financed by the Ministry of Health, to which annual reports are sent. 
